# Pickering Emulsions Stabilized by a Naturally Derived One-Dimensional All-In-One Hybrid Nanostructure

**DOI:** 10.1021/acs.langmuir.4c04712

**Published:** 2025-02-12

**Authors:** Yikai Feng, Chen Li, Haoran Jin, Yajuan Sun, Hang Jiang, Yunxing Li, To Ngai

**Affiliations:** † Key Laboratory of Synthetic and Biological Colloids, Ministry of Education, School of Chemical and Material Engineering, Jiangnan University, Wuxi 214122, China; ‡ Department of Chemistry, The Chinese University of Hong Kong, Shatin, N. T., Hong Kong 00852, China; § School of Chemistry, Biology and Environment, Yuxi Normal University, Yuxi 653100, China

## Abstract

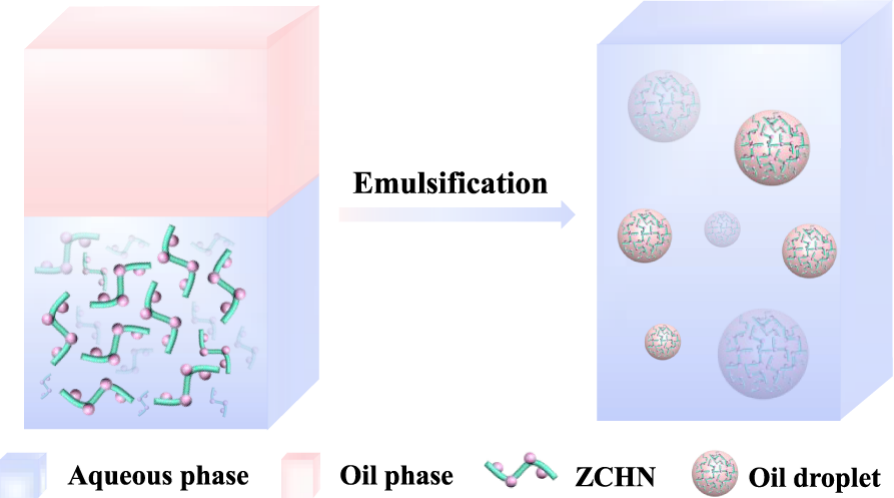

Colloidal particles generated from plant-derived proteins and polysaccharides have high potential as particulate stabilizers since they are environmentally friendly, biocompatible, and biodegradable. It has also been shown that amphiphilic anisotropic particles are more effective particulate stabilizers at the oil/water interface. In this study, a one-dimensional all-in-one zein nanoparticle/cellulose nanofiber hybrid nanostructure (ZCHN) was successfully prepared by self-assembling hydrophilic cellulose nanofibers (CNFs) and hydrophobic zein nanoparticles (ZNPs). The synthetic approach is based on the antisolvent-induced deposition of uniform and discrete ZNPs on the surface of CNFs, with the electrostatic interaction between the two thought to be the main factor for their binding. Furthermore, the microstructure of the generated ZCHN can be easily tuned by the initial mass ratio of zein and CNFs. When compared to ZNPs or CNFs alone or their simple mixture, the emulsion stabilized with ZCHN displayed better long-term, high-temperature, and centrifugation stability. The efficient reduction of oil/water interfacial tension, neutral wettability, and more uniform and high coverage of ZCHN on the droplet surface were the reasons for such better emulsion stability. As an illustration, the resulting emulsion protected β-carotene effectively, exhibiting a significant improvement in stability under UV radiation and high temperature. Therefore, the prepared biocompatible Pickering emulsion is anticipated to have promising applications for the preservation and delivery of fat-soluble bioactive compounds.

## Introduction

Pickering emulsions have a significant stability advantage over surfactant-stabilized emulsion, owing to the irreversible adsorption of particulate stabilizers at the biphasic interface.^1−4^ The desorption energy (Δ*E*) required to remove particles from the oil/water interface is substantially higher than thermal energy, leading to enhanced emulsion stability.^5^ Previous studies have found considerable differences in Δ*E* among particles with varied morphologies at the oil/water interface. One-dimensional nanostructured particles, such as rods, ellipsoids, and fibers, have higher adhesion energy than isotropic spherical particles.^1,6,7^ Furthermore, such nanostructures with high aspect ratios can intertwine with one another at the interface, resulting in more stable emulsions.^8,9^

The use of biopolymer-based particles as emulsion stabilizers has increased within the past ten years in order to fulfill the demands of food, pharmaceutical, and cosmetic industries.^10−15^ Among these, cellulose nanofibers (CNFs) derived from renewable cellulose are attractive candidates due to their one-dimensional morphology (high aspect ratio), flexibility, and self-assembling ability at the oil/water interface.^16−18^ However, they are difficult to adsorb at the oil/water interface because of their high hydrophilicity. Several approaches have been used to introduce hydrophobic sites, such as chemical reaction or physical adsorption; however, these treatments can degrade the natural attributes of CNFs.^19−21^ For example, Yuan et al. found that acetylation with acetic anhydride is an effective method for increasing the hydrophobicity of CNFs, and the resulting modified CNFs with amphiphilic characteristics can be used to prepare Pickering emulsions.^22^ Silva et al. found that stable Pickering emulsions could be prepared by cationizing CNFs with glycidyl trimethylammonium chloride.^23^

The use of binary particles as stabilizers to improve emulsion stability could be an effective strategy for avoiding unnecessary surface treatments that damage the original characteristic of CNFs.^24^ Different mechanisms have been proposed to explain the stabilization of the emulsions by binary particles. Binks et al. were the first to demonstrate that oppositely charged particles can stabilize Pickering emulsions. They proposed that the heteroaggregation of these particles is essential for forming stable emulsions, as it leads to aggregates with improved wettability.^25^ In contrast, our observation reveals a different stabilization mechanism, where positively charged zein nanoparticles (ZNPs) preferentially adsorb onto negatively charged oil droplets, followed by the adsorption of negatively charged starch nanocrystals, resulting in a bilayer coating of particles on the droplet surface.^26^ However, the use of binary particles is more complex than using a single particle stabilizer, since it requires optimal matching performance of the two particles, including mass ratios, particle interaction, and surface properties.^25,27−29^ When Pickering emulsions are prepared, the oil/water ratio, particle concentration, and emulsification procedures should all be considered, which will further increase the experimental workload. As a result, the preparation of all-in-one nanostructured hybrid particles based on CNFs is crucial for the development of high-performance Pickering stabilizers that can be directly added to the aqueous phase for emulsification experiments.

Controlling the surface morphology and composition of colloidal materials is an effective method for influencing their wettability.^30,31^ Zein is an alcohol-soluble protein derived from corn that is noted for its strong biocompatibility, biodegradability, and distinct hydrophobic properties.^32^ ZNPs generated from it have attracted great attention in emulsion preparation.^33^ To address current limitations, a simple and effective strategy for developing completely biopolymer-based Pickering emulsion stabilizers based on CNFs is essential. In this study, a one-dimensional, all-in-one hybrid nanostructure composed of ZNPs and CNFs, abbreviated as ZCHN, was constructed and used to prepare Pickering emulsions. In detail, ZNPs were coated onto CNFs using an antisolvent procedure, yielding a one-dimensional nanocomplex. The emulsification and stabilization mechanism of as-prepared ZCHN was further evaluated based on emulsion stability and interfacial behavior of ZCHN. Finally, using β-carotene as a model, the protective effect of Pickering emulsion on bioactive compounds was assessed.

## Experimental Section

### Materials

Cellulose nanofibers (CNFs) were supplied by Hangzhou Yuhan Science and Technology Co. Ltd. 2,2′-Azoisobutyronitrile (AIBN), zein, and pyrene were purchased from Sigma-Aldrich (China). Decamethylcyclopentasiloxane (D5) and β-carotene were bought from Aladdin Reagent Co., Ltd. (China) and Macklin Biochemical Technology Co., Ltd. (China), respectively. NaCl, NaOH, HCl, *n*-hexane, absolute ethanol, and styrene were supplied by Sinopharm Chemical Reagent Co., Ltd. (China). Fluorescein isothiocyanate (FITC), fluorescein sodium, and calcofluor white were supplied by TCI Development Co., Ltd. (China); Innochem Science & Technology Co., Ltd. (China); and Maokang Biotechnology Co., Ltd. (China), respectively. All the experiments were carried out using deionized water.

### Preparation of Zein Nanoparticles (ZNPs)

1.5 g of zein powder was first dissolved in aqueous ethanol (40 mL, 75 vol %) under stirring. Following that, the prepared zein solution was mixed with water (120 mL) under stirring. After rotary evaporation under reduced pressure, ethanol and some water were removed, yielding a concentrated dispersion of ZNPs that was stored at 4 °C until it was used.

### Preparation of Zein Nanoparticles/Cellulose Nanofiber Hybrid Nanostructure (ZCHN)

In 40 mL of aqueous ethanol, CNFs (0.2 g) and a certain quantity of zein powder were mixed under stirring. Then, water (120 mL) was injected into the aforementioned mixture under stirring. The resultant ZNPs deposited on the surface of CNFs, namely, ZCHN. Using vacuum rotary evaporation, the ethanol and some of water were eliminated. The resulting particle dispersions were stored at 4 °C until they were used. The initial mass ratios of zein to CNFs were 5:1, 2:1, and 1:1, respectively.

### Confocal Laser Scanning Microscopy (CLSM)

A Nikon Eclipse Ti inverted microscope (Japan) equipped with laser lines at 405 and 488 nm was used to take CLSM pictures. Calcofluor white and FITC were used to stain the CNFs and ZNPs of ZCHN, respectively. Specifically, CNFs were mixed with calcofluor white (0.01 wt %) for 12 h before emulsification, while FITC (0.01 wt %) was added to the aqueous ethanol during ZNP preparation for labeling.

### Scanning Electron Microscopy (SEM)

SEM images were acquired by using a Hitachi S-4800 scanning electron microscope (Japan). The aqueous dispersion of particles (0.1 wt %) was deposited on the silicon wafer. After drying at room temperature, the sample was coated with a thin layer of gold before being examined under SEM.

### Zeta Potential Measurements

The zeta potential was measured using a Zeta PALS analyzer (Brookhaven, USA). The aqueous dispersions of CNFs and ZNPs were each diluted 100-fold with water, and the pH was adjusted with NaOH (0.1 M) or HCl (0.1 M). Each sample was measured in triplicate at 25 °C.

### Three-Phase Contact Angle (θ_ow_) Measurements

The three-phase contact angle (θ_ow_) of particulate stabilizers was acquired with an OCA 15EC optical contact angle video system (DataPhysics, Germany). The powdery sample was compressed into a thin tablet before being immersed in oil. A water droplet was then placed on each tablet by using a syringe. The software calculated the θ_ow_ after a camera recorded an image of the water droplet. Each experiment was repeated at least three times.

### Dynamic Interfacial Tension (IFT) Measurements

The dynamic interfacial tension of the oil/water interface was recorded using a pendant water droplet in a bulk phase of oil. An OCA 15EC optical contact angle video system (DataPhysics, Germany) was used for the measurements. After being mounted at the end of an injector filled with an aqueous dispersion of particles, an injection needle was inserted in a cuvette filled with oil. Then, the aqueous phase was squeezed into the oil gradually. The dynamic IFT was measured using continuously recorded droplet pictures collected at room temperature. The measurements were taken three times.

### Preparation and Characterization of Pickering Emulsions

Particulate stabilizers were diluted with water to achieve a particle dispersion (1 wt %). The emulsion was then prepared after the particle dispersion was homogenized for 2 min at 17 000 rpm with an equal volume of oil. The emulsion microstructure was examined by using a Nikon Eclipse Ti inverted microscope (Japan) and a Leica DM 500 optical microscope (Germany). The average droplet size was estimated by measuring the diameter of at least 300 emulsion droplets in each emulsion by using ImageJ software. CLSM images were acquired with the oil and aqueous phases colored with perylene and fluorescein sodium, respectively. The CNFs and ZNPs were stained with calcofluor white and FITC, respectively.

### Evaluation of Emulsion Stability

The storage stability was assessed by tracking changes in the droplet size and appearance at room temperature. The degree of oil separation following centrifugation at 6000 *g* for 10 min (Eppendorf 5804R, USA) was used to assess their centrifugation stability. The Pickering emulsions prepared with various particulate stabilizers were stored at 80 °C for 24 h to evaluate their high-temperature stability. The microstructure and visual appearance of each emulsion were analyzed.

### Visualization of the Emulsion Microstructure with SEM

Polystyrene (PS) microspheres were synthesized by the solidification of styrene droplets stabilized with two types of particulate stabilizers. In detail, styrene dissolved with AIBN (1 wt %) and an aqueous dispersion of the particulate stabilizer were combined at a volume ratio of 1:9. The emulsion was prepared by homogenizing the above mixture for 2 min at 17 000 rpm. The emulsion was then polymerized at 60 °C for 24 h. After returning to room temperature, the resultant product was collected via centrifugation and redispersion cycles. After being coated with a thin gold overlayer, the dried sample was observed with SEM.

### Protection of β-Carotene

β-Carotene (0.1 wt %) was dissolved in oil under ultrasonication, followed by centrifugation to remove any undissolved β-carotene. A Pickering emulsion containing β-carotene was then prepared by using oil dissolved with β-carotene. The control group was a β-carotene oil solution. The stability of β-carotene was then tested under different environmental conditions. For the influence of temperature, the resulting emulsion containing β-carotene was kept in the dark at 50 °C. To test the influence of UV radiation at room temperature, emulsions in glass bottles were exposed to UV light (250 W/m^2^). Periodic measurements were taken to monitor the retention of β-carotene. Each sample (0.2 g) was mixed with ethanol (1 mL) and *n*-hexane (2 mL) and then vortexed in order to extract β-carotene. The *n*-hexane layer was collected, and the total volume was adjusted to 10 mL after two extractions. At 450 nm, the absorbance was measured. The retention rate was calculated as follows:
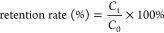
where β-carotene concentrations before and after storage were denoted by *C*_0_ and *C*_t_, respectively.

## Results and Discussion

### Preparation of a One-Dimensional All-In-One Hybrid Nanostructure

**Figure 1:**
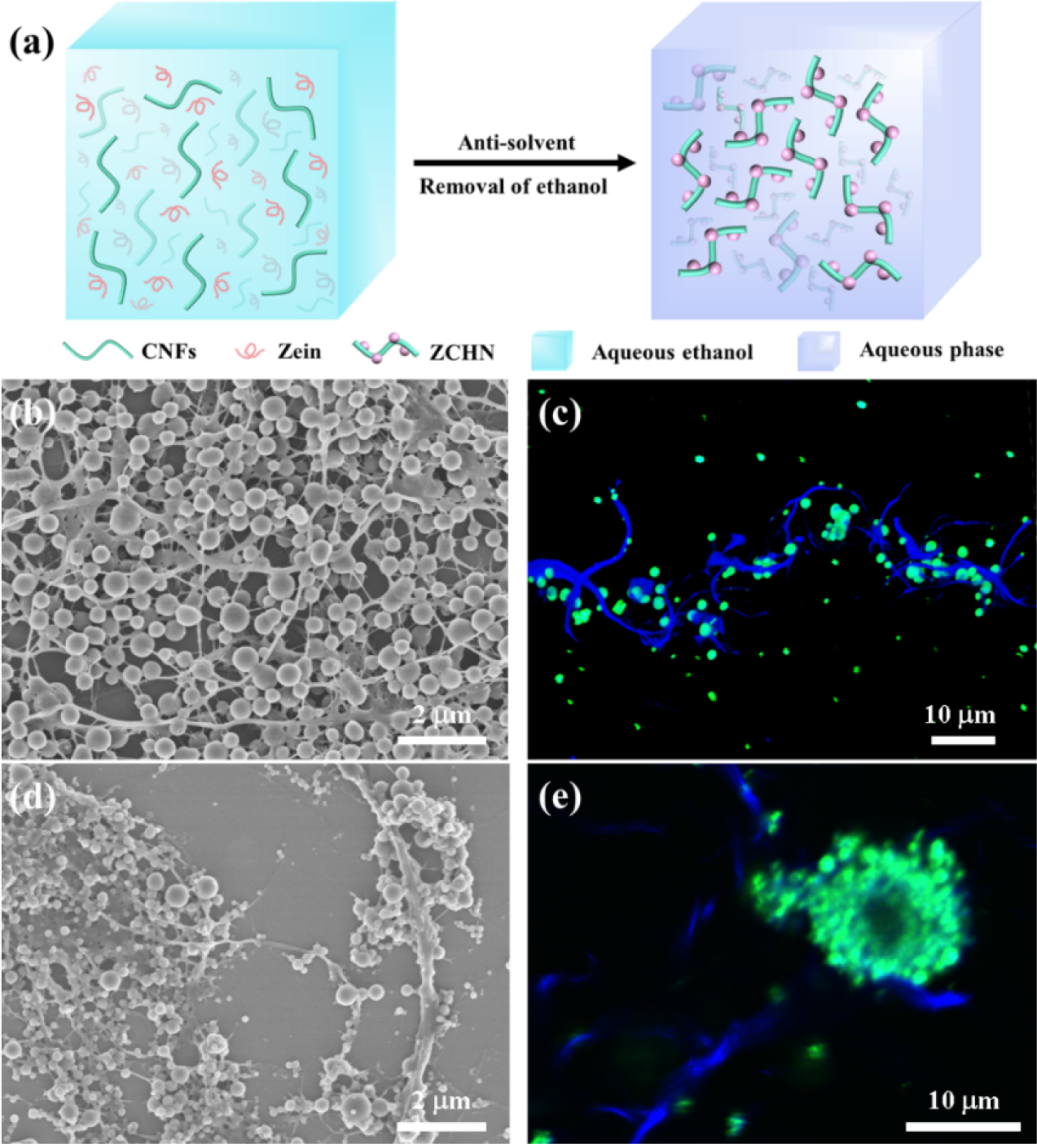
(a) Schematic illustration of the preparation of ZCHN. SEM and CLSM images of ZCHN (b, c) and ZCDA (d, e).

As illustrated in Figure 1a, a one-dimensional all-in-one zein nanoparticle/cellulose nanofiber hybrid nanostructure (ZCHN) was prepared facilely in the dispersion of CNFs using in situ attachment of ZNPs through an antisolvent procedure.^34^ In detail, CNFs were dispersed in aqueous ethanol, followed by the addition and dissolution of zein powder under constant stirring. While the mixing was continued, water was added to decrease the solubility of zein. As the solubility of zein decreased, zein started to precipitate and attach to the surface of CNFs. The morphology of pristine CNFs was examined using SEM, and they were easily bendable and showed a high aspect ratio with diameters less than 100 nm and lengths ranging from a few to tens of micrometers (Figure S1). SEM and CLSM images of the representative ZCHN revealed that ZNPs with relatively uniform size (ca. 370 nm) were discretely immobilized along the CNFs (Figure 1b,c). The composition of ZCHN was further examined using FTIR spectroscopy. In Figure S2, the FTIR spectra revealed distinctive absorption peaks corresponding to ZNPs and CNFs, indicating that the ZCHN was composed of both. When observed with CLSM, CNFs and ZNPs were labeled with calcofluor white and FITC, respectively. For comparison, free ZNPs were first prepared using the antisolvent method in advance (Figure S1). In contrast, Figure 1d,e displays that when as-prepared ZNPs and CNFs were mixed directly in water, the product was a kind of disordered aggregate composed of ZNPs and CNFs (ZCDA) and generated by the random arrangement of the two particles.

### Formation Mechanism of the One-Dimensional All-In-One ZCHN

**Figure 2:**
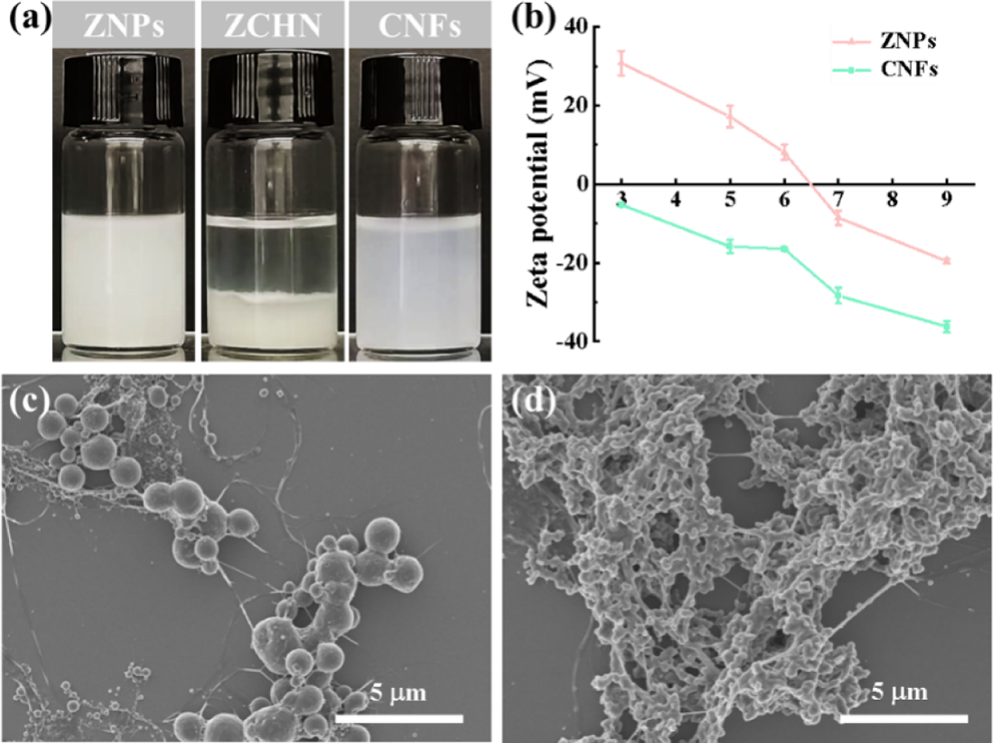
(a) Digital images of aqueous dispersions of ZNPs, ZCHN, and CNFs. (b) Zeta potential of ZNPs and CNFs at various pH levels. SEM images of ZCHN prepared at pH values of 6 (c) and 9 (d), respectively.

Figure 2a displays the appearance of aqueous dispersions of ZNPs, CNFs, and ZCHN, following 1 day of preparation. The difference in the visual inspection of these dispersions also clearly indicated the formation of ZCHN. Compared to the dispersions of ZNPs and CNFs, ZCHN was expected to have a larger effective particle size and weight, leading to more rapid sedimentation within the same time. As shown in Figure 1b,c, ZCHN was prepared using the antisolvent procedure of zein at a pH of 3 in the presence of CNFs. Figure 2b depicts the zeta potentials of CNFs and ZNPs at various pH levels. CNFs appeared to have a negatively charged surface in all pH ranges evaluated. The zeta potential of CNFs decreased as the pH decreased. As for ZNPs, it was positively charged when the pH was below its isoelectric point, and when the pH was above its isoelectric point, it was negatively charged. Consistent with previous studies, its isoelectric point was close to 6.^32^ Therefore, the generated ZNPs have opposite charges to CNFs at a pH of 3, and the formation of well-defined ZCHN can be due to the electrostatic interaction between the two particles.

To verify the role of electrostatic interaction in the preparation of ZCHN, the antisolvent procedures of zein were also carried out at pH values of 6 and 9 in the presence of CNFs, respectively. Figure 2c,d shows representative SEM images of the as-produced samples. When the pH was elevated to 6, quasi-spherical ZNPs could still be obtained; however, due to being near the isoelectric point, the formed ZNPs were unstable, substantial agglomeration developed between the particles, and the resulting products were irregular aggregates composed of CNFs and ZNPs. At a pH of 9, both CNFs and the ZNPs formed had negative charges. The SEM image revealed that the microstructure of the prepared sample was more disordered, while the morphology of particulate zein was more irregular. These findings add to the evidence that the formation of ZCHN is dependent on the surface electrical properties of ZNPs and CNFs.

### Comparison of the Emulsifying Ability of Different Particulate Stabilizers

**Figure 3:**
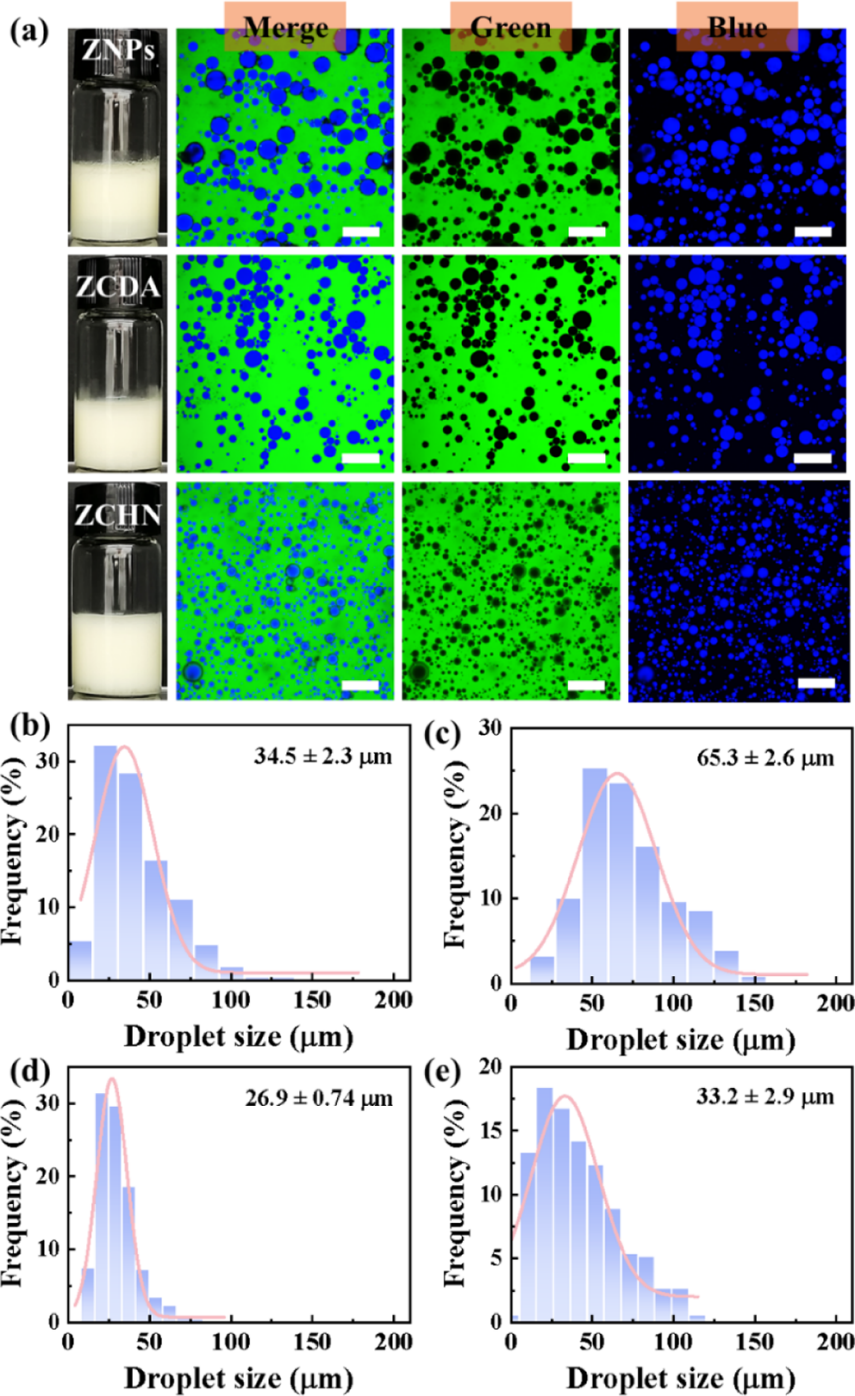
(a) Digital images and CLSM images of fresh emulsions prepared with ZNPs, ZCDA, and ZCHN, respectively; statistical droplet size distributions of emulsions stabilized with ZCDA (b, c) and ZCHN (d, e) at room temperature, both freshly prepared and one month later. The oil phase volume fraction was 50 vol %, and the concentration of particulate stabilizers was 1 wt %. Every scale bar is 200 μm.

Pickering emulsions were prepared by employing a variety of particulate stabilizers, including ZNPs, CNFs, ZCHN, and ZCDA. Their stability was assessed using the change in emulsion appearance and microstructure with time. When CNFs were used in the oil/water system, appreciable phase separation occurred after emulsification (Figure S3). Figure 3a shows that all other particles successfully emulsified the oil/water mixture. The microstructure of these emulsions was further examined using CLSM. Perylene was used to color the oil (blue), while the aqueous phase (green) was dyed with fluorescein sodium. As seen from these images, the type of all emulsions was oil-in-water. Compared to emulsions stabilized with ZNPs and ZCDA, the resulting emulsion using ZCHN as the stabilizer had smaller droplets and narrower droplet size distribution. In general, emulsion stability increases with its smaller and narrower droplet size distribution. Indeed, the ZNP-stabilized emulsion showed appreciable oil leakage after just 3 days (Figure S4). Furthermore, when the storage period was extended to one month, the droplet size increased obviously when the emulsion was stabilized with ZCDA, but not when stabilized with ZCHN (Figures S5, S6, and 3).

**Figure 4:**
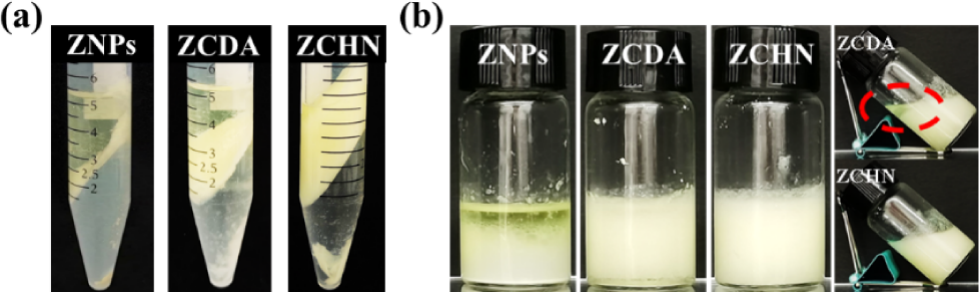
Visual appearance of emulsions stabilized with ZNPs, ZCDA, and ZCHN following centrifugation at 6000 *g* for 10 min (a) and after 24 h of storage at 80 °C (b). Oil phase volume fraction was 50 vol %, and the concentration of particulate stabilizers was 1 wt %.

The stability of these emulsions was then assessed under centrifugation and high temperature. As demonstrated in Figure 4a, the emulsion prepared with ZCHN remained the most stable under the same centrifugation conditions. In contrast, the ZCDA-stabilized emulsion separated a significant amount of oil. Even worse, the ZNP-stabilized emulsion showed nearly complete phase separation. Centrifugation can be used to accelerate the creaming and coalescence of the emulsion droplets. The high stability of the ZHCN-stabilized emulsion can be attributed to its smaller droplet size. The emulsion creaming slowed, and the collision probability of emulsion droplets decreased, thus limiting the droplet coalescence and oil separation. On the other hand, the emulsion stabilized by ZCHN also remained relatively stable at high temperature, with just a certain increase in droplet size following heating treatment (Figure S7). However, the other two emulsions showed appreciable oil leakage (Figure 4b). In a word, the ZCHN had better a emulsifying ability, and the Pickering emulsion prepared from it was more stable under various environmental stresses. It is also noteworthy that the emulsifying ability of ZCHN could be altered by its microstructure. The amount of ZNPs supported on the CNFs reduced as the zein concentration decreased during the preparation of ZCHN (Figure S8). At the same time, the droplet size of prepared emulsions with the resulting ZCHN increased (Figure S9).

### Stabilization Mechanism of Pickering Emulsion Prepared with ZCHN

**Figure 5:**
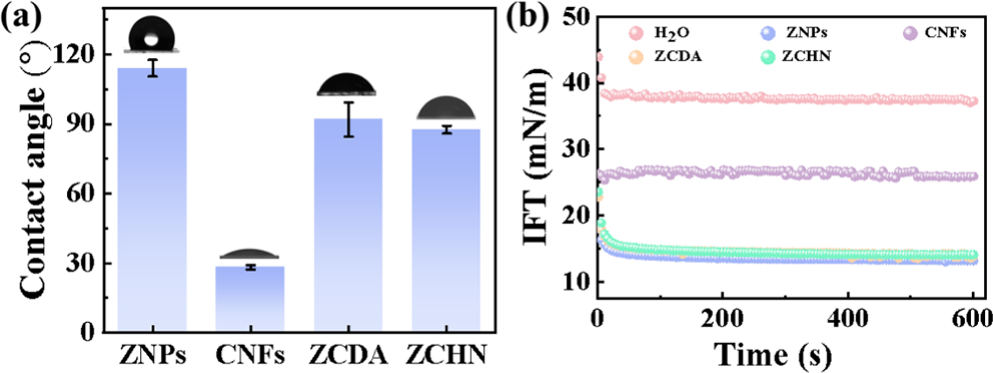
(a) θ_ow_ of the water droplet on the tablets of ZNPs, CNFs, ZCDA, and ZCHN immersed in the oil phase. (b) Dynamic interfacial tension of oil/water interfaces with or without particulate stabilizers.

The ZCHN-stabilized emulsion was more stable than those prepared by using ZNPs, CNFs, and ZCDA as stabilizers. To understand the stabilizing mechanism, the interfacial behavior of particulate stabilizers and the emulsion microstructure were explored. The surface wettability of particles influences their emulsifying ability, which may be reflected using the three-phase contact angle (θ_ow_). As illustrated in Figure 5a, ZNPs and CNFs were unable to successfully stabilize the emulsion due to their high hydrophobicity or hydrophilicity, as seen by their θ_ow_ values of 114° and 28°, respectively. In contrast, the θ_ow_ values of ZCHN and ZCDA were close to 90°, falling between those of individual ZNPs and CNFs. Clearly, these particles had almost equal affinity for water and oil that constituted the interface. In general, neutral wettability can maximize the stabilizing ability of particles. Nonetheless, ZCHN had more superior nanostructured topography and dispersity, as observed previously, which may further improve its emulsifying ability. On the other hand, emulsions are multiphase systems with an enormous interface area, and lowering the interfacial tension is critical for emulsion stability. Figure 5b shows that ZNPs, ZCDA, and ZCHN lowered the oil/water interfacial tension more efficiently than CNFs. The oil/water interfacial tension in the absence of particulate stabilizers was about 37.5 mN/m. When CNFs were introduced to water, the interfacial tension decreased to 26.7 mN/m.^35,36^ In contrast, the addition of ZNPs, ZCDA, or ZCHN reduced the interfacial tension to less than about 14.5 mN/m. Moreover, it is worth noting that in these three cases, after a quick fall, the interfacial tension gradually decreased, possibly due to particle reorganization at the interface.^37^ These results showed that these four types of particles could act as barriers at the interface that stabilized droplets. However, unlike ZCHN and ZCDA, CNFs and ZNPs alone cannot successfully maintain the emulsion stabilization because they were either too hydrophilic or hydrophobic.

**Figure 6:**
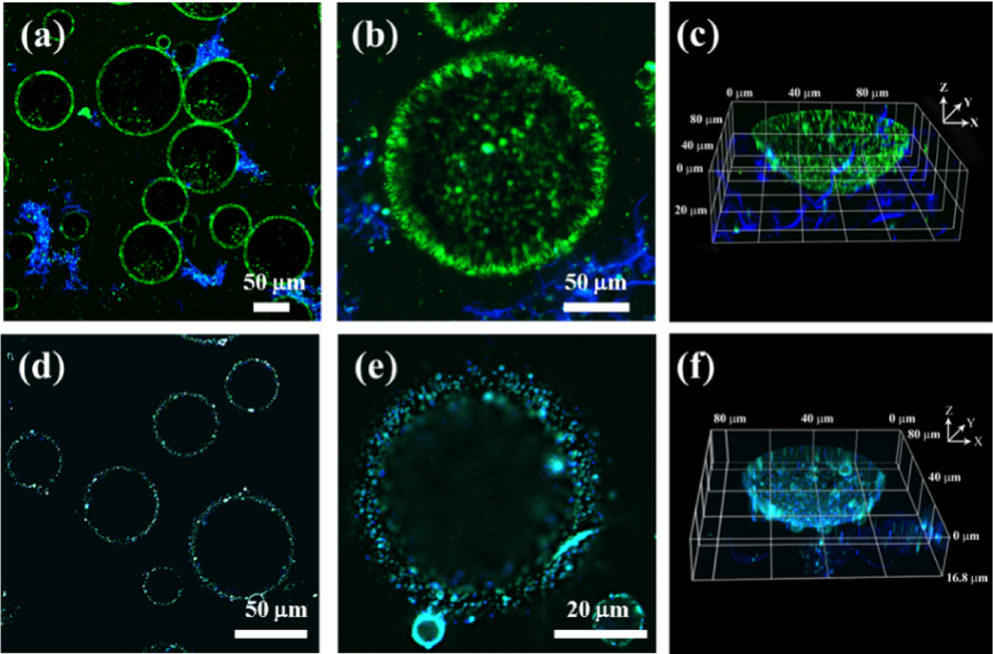
CLSM images of Pickering emulsions stabilized with ZCDA (a–c) and ZCHN (d–f) with low and high magnifications as well as their 3D reconstructed images.

Given that ZCHN and ZCDA had similar wettability and interfacial activity, microscopic structural analyses with CLSM were performed to further explore the variation in the stability of emulsions prepared with them. This technique was chosen because it enabled intuitive observations of the emulsion microstructure under in situ situations. Figure 6 shows CLSM images of ZCHN and ZCDA-stabilized emulsions that had been fluorescently tagged. The blue and green patches around the droplets correspond to dye-labeled CNFs and ZNPs, respectively. Figure 6a,d shows that both ZCHN and ZCDA-stabilized emulsions had considerable adsorption of particles at the interface, showing that the particle barrier could prevent droplet coalescence. However, in the ZCDA-stabilized emulsion, the droplet surface appeared to be attached with ZNPs more easily, whereas CNFs preferentially dispersed in water (Figure 6a–c). In contrast, when ZCHN was used as the particulate stabilizer, both ZNPs and CNFs were concentrated at the surface of emulsion droplets (Figure 6d–f). Additionally, CLSM images also showed that the CNF bridges held some emulsion droplets together (Figure S10).

**Figure 7:**
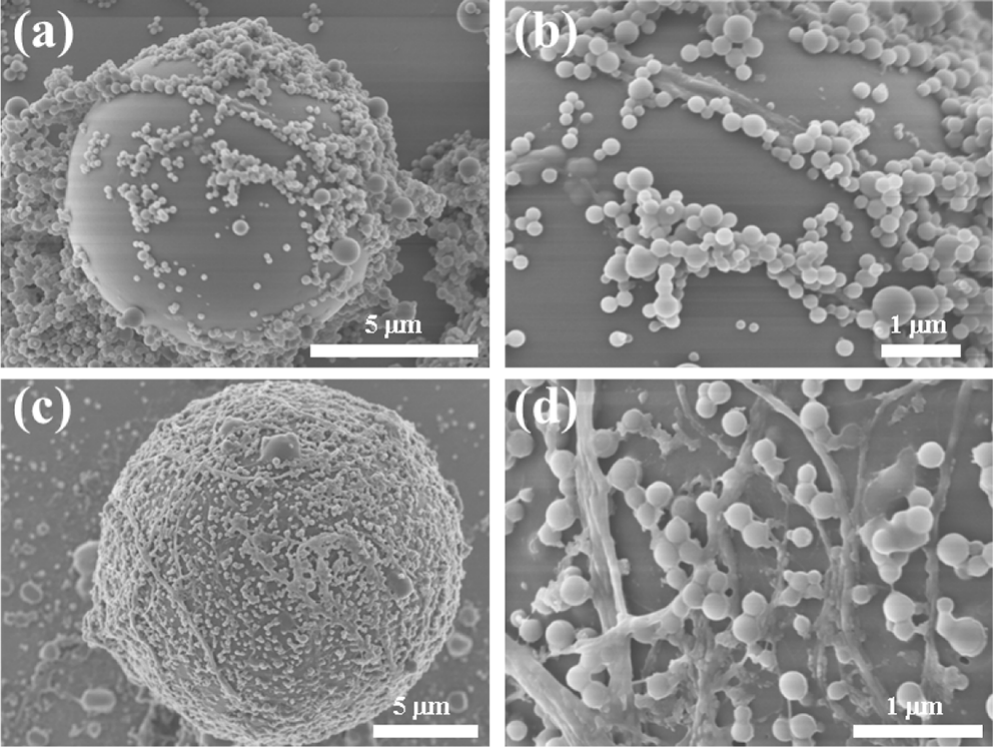
SEM images of polymerized styrene droplets prepared with ZCDA (a, b) and ZCHN (c, d) with low and high magnifications.

Although CLSM can validate the positions of ZCHN and ZCDA within the emulsions, it cannot clearly represent the microstructures they constructed at the interface owing to the nanoscale size of the CNFs and ZNPs. In comparison with CLSM, SEM has a higher magnification and resolution, making it suitable for observing nanoscale objects. Because conventional SEM cannot be used to image liquid emulsions, a method was developed in which styrene was used as the oil phase and polymerized, allowing to track the location of ZCHN and ZCDA in the Pickering emulsions.^38^ In Figure 7a,b, for the emulsion prepared with ZCDA, the particles at the oil/water interface were sparse, with ZNPs dominating, and there was significant aggregation. Therefore, the discontinuous particle adsorption layer on the droplet surface and the possible network generated by ZCDA in the aqueous phase (Figure 6a), provided the emulsion with stability. In contrast to ZCDA, the surface of droplets was almost fully enveloped by ZCHN, resulting in a dense and uniform adsorption layer of particles (Figure 7c). A SEM image with higher magnification clearly shows that this interfacial layer consists of interconnecting CNFs and ZNPs (Figure 7d). This explained how the emulsion remained stable over time, and under diverse environmental stresses, the dense adsorption of ZCHN at the oil/water interface enabled emulsion droplets to be more stable.

Therefore, the high stability of ZCHN-stabilized emulsion could be explained as follows: on the one hand, both the capacity of ZCHN to reduce the oil/water interfacial tension and its intermediate wettability were advantageous for emulsion formation with smaller droplets; on the other hand, the dense and uniform interfacial adsorption layer of the ZCHN serving as a physical barrier prevented droplets from coalescence, increasing the emulsion stability even more.

### Protection of β-Carotene in Pickering Emulsion

**Figure 8:**
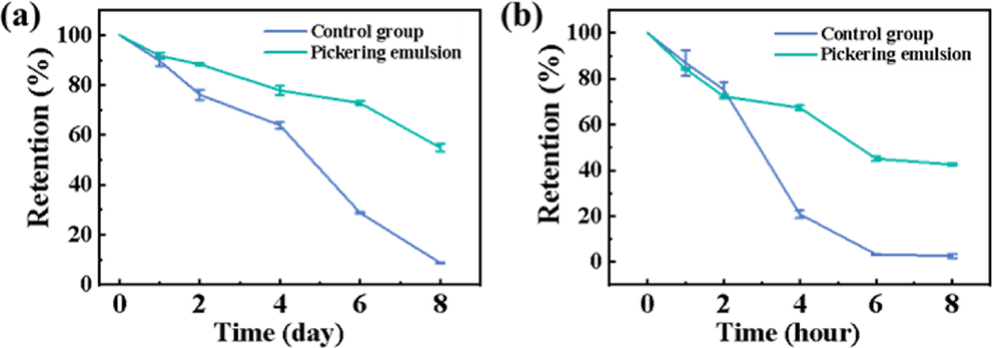
Retention of β-carotene in ZCHN-stabilized Pickering emulsions and its oil solution (a) at 50 °C and (b) under UV radiation.

Pickering emulsions have great stability and loading capacity, making them ideal for the protection of fat-soluble bioactive substances like β-carotene. This study compares the storage stability of β-carotene in its oil solution (control group) with that of ZCHN-stabilized Pickering emulsions. As shown in Figure 8a, after 8 days of heating at 50 °C, the retention of β-carotene in the former group was approximately 8.75%, while in the latter group, it had a higher retention of about 54.91%. Figure 8b shows that after 8 h of UV exposure, the retention of β-carotene in its oil solution dramatically decreased to about 2.49%. However, the retention of β-carotene rose dramatically in ZCHN-stabilized Pickering emulsion, reaching approximately 42.56%. Obviously, the Pickering emulsion was superior to bulk oil in terms of reduction of the degradation of this lipophilic active compound. Several factors contributed to this result.^39−41^ The dense particle adsorption layer around the oil droplets acted as a superior physical barrier, preventing β-carotene from accessing pro-oxidants in the aqueous phase, while it also increased the emulsion stability. Moreover, the presence of both negatively charged CNFs and positively charged ZNPs on the droplet surface could help prevent transition metal ions from interacting with β-carotene. Therefore, the one-dimensional all-in-one ZCHN developed in this study effectively stabilizes the oil–water interface, allowing for better protection of lipophilic active compounds, which has significant advantages for food, cosmetic, and pharmaceutical formulations.

## Conclusion

In summary, we present a new type of all-natural biopolymer-based particulate stabilizer. The adsorption of ZNPs on the surface of CNFs was induced by electrostatic interaction, resulting in a 1D all-in-one hybrid nanostructure. The successful preparation of such a structure was directly visualized using SEM and CLSM. Furthermore, the microstructure of ZCHN could be controlled by the initial zein/CNF mass ratio. The results of the three-phase contact angle and dynamic interfacial tension revealed that ZCHN exhibited good interfacial activity and moderate interfacial wettability. Compared to ZNPs, CNFs, and ZCDA, they had a better effective emulsification ability. In addition, as evidenced by SEM and CLSM, the ZCHN can form a network structure with high coverage at the oil–water interface, leading to more stable Pickering emulsions. Finally, encapsulating β-carotene in ZCHN-stabilized Pickering emulsion enhanced its stability under high temperature and UV radiation when compared to free β-carotene. The retention was about 6 and 17 times higher than those of the control groups, respectively. As a result, the proposed hybrid strategy will play an important role in the design and preparation of biocompatible and promising particulate stabilizers, which will guide the future development of food, cosmetics, and pharmaceuticals.

## Supporting Information Available

Microscopic images and FTIR spectra of various Pickering emulsion stabilizers; macroscopic and microscopic images of Pickering emulsions prepared with various Pickering emulsion stabilizers (PDF)
