# Supplementary material for: Pickering Emulsions Stabilized by a Naturally Derived One-Dimensional All-In-One Hybrid Nanostructure
Source: Langmuir. 2025 Feb 12;41(7):4748–55. doi: 10.1021/acs.langmuir.4c04712 (PMC11866913; doi:10.1021/acs.langmuir.4c04712)
Supplement: Supplementary file 1 — la4c04712_si_001.pdf [file la4c04712_si_001.pdf]

SUPPORTING INFORMATION

Pickering emulsions stabilized by naturally derived one-dimensional all-in-one hybrid nanostructure

Yikai Feng[†,⊥], Chen Li[†,§,⊥], Haoran Jin[†], Yajuan Sun[†], Hang Jiang[†], Yunxing Li[†*] and To Ngai[‡*]

[†] *Key Laboratory of Synthetic and Biological Colloids, Ministry of Education, School of Chemical and Material Engineering, Jiangnan University, Wuxi 214122, China*

[‡] *Department of Chemistry, The Chinese University of Hong Kong, Shatin, N. T. Hong Kong, China*

[§]*School of Chemistry, Biology and Environment, Yuxi Normal University, Yuxi 653100, China*

[⊥]*These authors contribute equally.*

* Corresponding author

Email: yunxingli@jiangnan.edu.cn (Y. X. Li); tongai@cuhk.edu.hk (T. Ngai)

Number of pages: 6

Number of figures: 10

Table of Contents

Figure S1. SEM images of CNFs and ZNPs.

Figure S2. FTIR spectra of ZNPs, CNFs and ZCHN.

Figure S3. Appearance of the fresh emulsion prepared with CNFs.

Figure S4. Appearance of the ZNPs-stabilized Pickering emulsion after 3 days.

Figure S5. Optical images of Pickering emulsions stabilized with ZCDA (a and b) and ZCHN (c and d) at room temperature, both freshly prepared and one month later. Scale bars are 200 μm.

Figure S6. Statistical average droplet sizes of emulsions stabilized with ZCDA and ZCHN at room temperature, both freshly prepared and 1 month later. (*p < 0.05 and ***p < 0.001, fresh vs 1 month).

Figure S7. CLSM images of ZCHN-stabilized Pickering emulsions following new preparation (a) and storage at 80 ºC for 24 h (b). Scale bars are 200 μm.

Figure S8. SEM images of ZCHN prepared with different initial mass ratios between zein and CNFs: (a) 5:1, (b) 2:1, and (c) 1:1. Scale bars are 10 μm.

Figure S9. Optical images of Pickering emulsions stabilized by ZCHN prepared with different initial mass ratios between zein and CNFs: (a) 5:1, (b) 2:1, and (c) 1:1. Scale bars are 200 μm.

Figure S10. CLSM image of the ZCHN-stabilized Pickering emulsion.

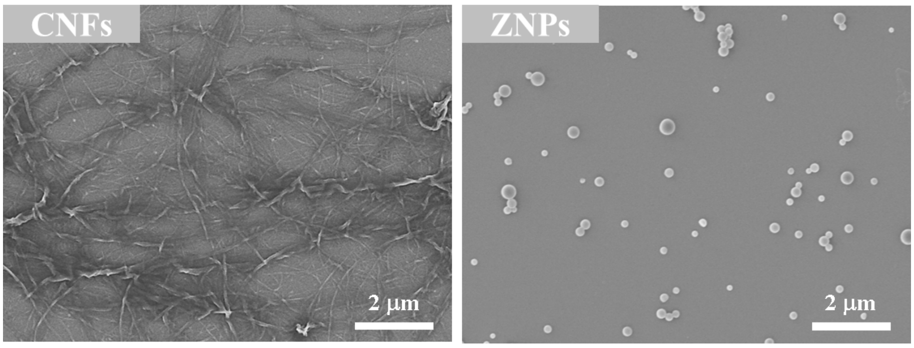

Figure S1. SEM images of CNFs and ZNPs.

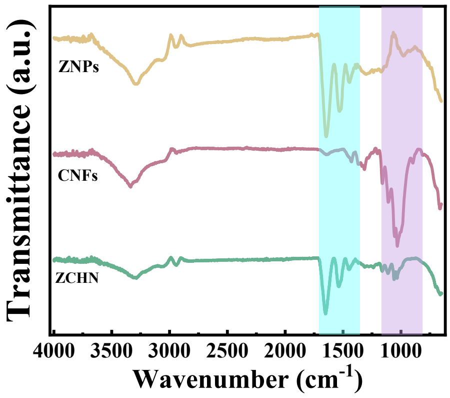

Figure S2. FTIR spectra of ZNPs, CNFs, and ZCHN.

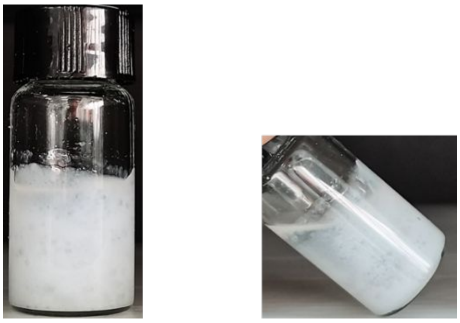

Figure S3. Appearance of the fresh emulsion prepared with CNFs.

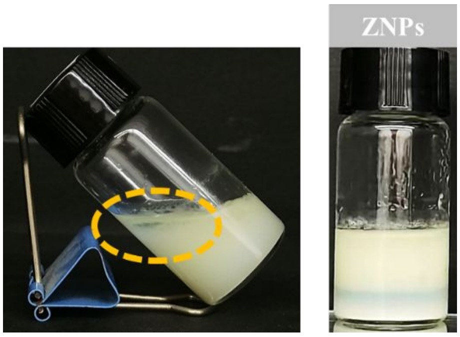

Figure S4. Appearance of the ZNPs-stabilized Pickering emulsion after 3 days.

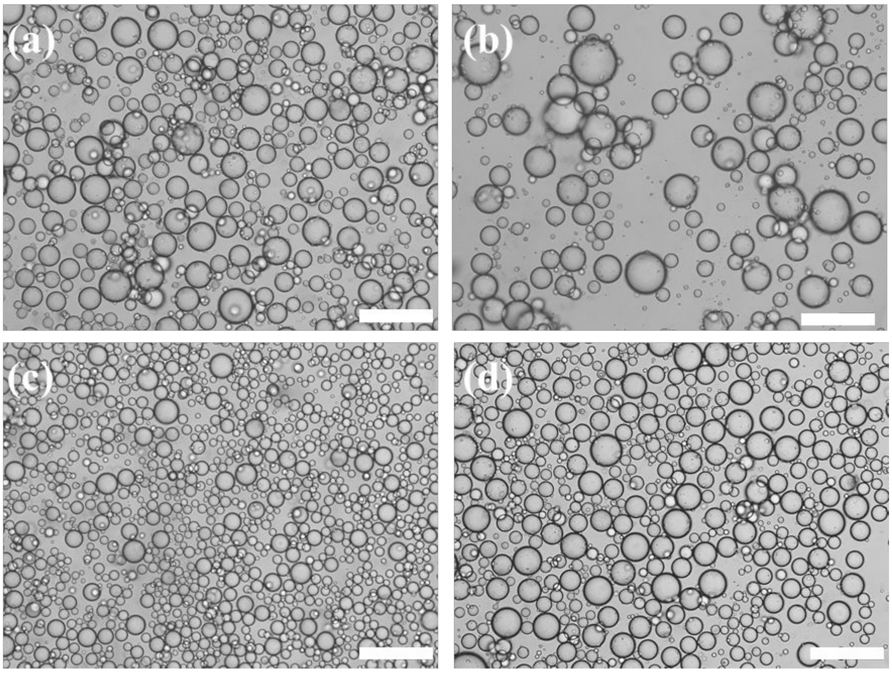

Figure S5. Optical images of Pickering emulsions stabilized with ZCDA (a and b) and ZCHN (c and d) at room temperature, both freshly prepared and one month later. Scale bars are 200 μm.

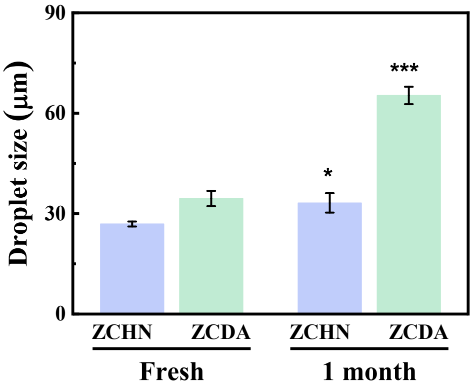

Figure S6. Statistical average droplet sizes of emulsions stabilized with ZCDA and ZCHN at room temperature, both freshly prepared and 1 month later. (*$p < 0.05$ and ***$p < 0.001$, fresh vs 1 month).

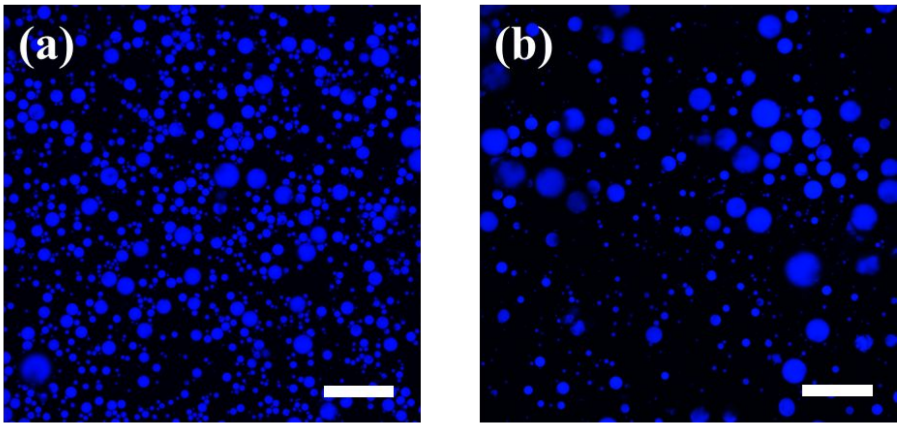

Figure S7. CLSM images of ZCHN-stabilized Pickering emulsions following new preparation (a) and storage at 80 ºC for 24 h (b). Scale bars are 200 μm.

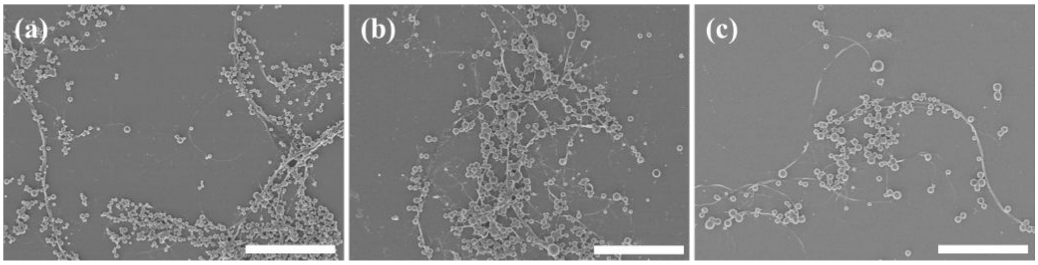

Figure S8. SEM images of ZCHN prepared with different initial mass ratios between zein and CNFs: (a) 5:1, (b) 2:1, and (c) 1:1. Scale bars are 10 μm.

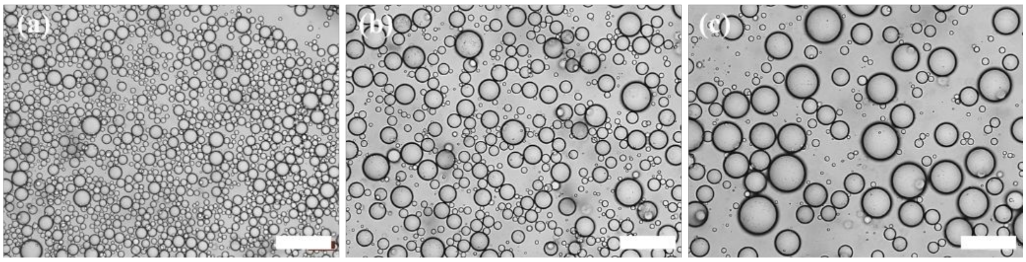

Figure S9. Optical images of Pickering emulsions stabilized by ZCHN prepared with different initial mass ratios between zein and CNFs: (a) 5:1, (b) 2:1, and (c) 1:1. Scale bars are 200 μm.

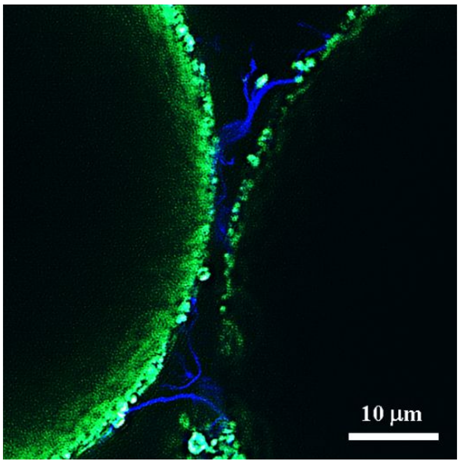

Figure S10. CLSM image of the ZCHN-stabilized Pickering emulsion.
